# Association of Global Longitudinal Strain With Left Ventricular Remodeling and Left Ventricular Hypertrophy in Patients With Acute Myocardial Infarction After PCI: A Small Single‐Center Prospective Observational Study

**DOI:** 10.1002/hsr2.72716

**Published:** 2026-06-28

**Authors:** Bing Wang, Hong Yao, Zhijian Zhu, Zhigang Lu, Yesheng Pan

**Affiliations:** ^1^ Department of Cardiology Jinshan Branch of Shanghai Sixth People's Hospital Shanghai China; ^2^ Department of Ultrasound Jinshan Branch of Shanghai Sixth People's Hospital Shanghai China; ^3^ Department of Cardiology Shanghai Jiao Tong University Affiliated Sixth People's Hospital Shanghai China

**Keywords:** acute myocardial infarction, global longitudinal strain, left ventricular hypertrophy, left ventricular remodeling

## Abstract

**Background and Aims:**

Among patients with acute myocardial infarction undergoing PCI, subsequent structural alterations of the left ventricle, including left ventricular remodeling (LVR) and left ventricular hypertrophy (LVH), may contribute to later heart failure. This study examined whether GLS measured early after reperfusion could help identify patients at increased risk for these post‐infarction changes.

**Methods:**

A total of 131 patients with AMI who underwent primary PCI at our hospital between March 2023 and October 2024 were included. GLS was assessed within 7 days after PCI using two‐dimensional speckle‐tracking echocardiography. The primary endpoints were LVR and LVH assessed at 6–12 months of follow‐up. We used logistic regression to estimate the associations of GLS with the endpoints, applied restricted cubic splines (RCS) to explore possible nonlinear trends, and conducted subgroup analyses to assess the robustness of the results.

**Results:**

Logistic regression analyses demonstrated that impaired GLS was independently associated with a higher risk of LVR. In tertile analyses, compared with patients in T1, representing better preserved LV systolic function, those in T3, representing more impaired LV systolic function, had significantly higher risks of LVR (OR = 7.51, 95% CI: 1.41–39.96, *p* = 0.02) and LVH (OR = 6.15, 95% CI: 1.40–27.04, *p* = 0.02). RCS analysis indicated linear associations between GLS and the risks of both LVR and LVH. ROC analysis showed that the optimal GLS cut‐off values for predicting LVR and LVH were −11.54% and −11.22%, with AUCs of 0.724 and 0.774, respectively.

**Conclusion:**

Early impairment of GLS in patients with AMI after PCI was associated with subsequent LVR and LVH, suggesting that GLS may be useful for early risk stratification in this population.

**Abbreviations**
AMI: acute myocardial infarction
BMI: body mass index
CI: confidence interval
CKMB: creatine kinase MB isoenzyme
D2B: door‐to‐balloon time
DBP: diastolic blood pressure
eGFR: estimated glomerular filtration rate
GLS: global longitudinal strain
HDL‐C: high density lipoprotein‐cholesterol
hs‐CRP: high‐sensitivity C‐reactive protein
hs‐cTnT: high‐sensitivity cardiac troponin T
LAD: left anterior descending
LCX: left circumflex branch
LDL‐C: low density lipoprotein‐cholesterol
LVEF: left ventricular ejection fraction
LVH: left ventricular hypertrophy
LVR: left ventricular remodeling
MACE: major adverse cardiovascular events
NT‐proBNP: N‐terminal pro‐brain natriuretic peptide
OR: odds ratio
PCI: percutaneous coronary intervention
pre‐AP: pre‐infarction angina
RCA: right coronary artery
SBP: systolic blood pressure
SBT: symptom onset‐to‐balloon time
STE: speckle‐tracking echocardiography

## 1 Introduction

Although the widespread use of percutaneous coronary intervention (PCI) has improved survival after acute myocardial infarction (AMI), many patients continue to experience adverse changes in cardiac structure and function, which contribute to the persistent burden of post‐infarction heart failure [1, 2]. Among the structural abnormalities that contribute to adverse outcomes after AMI, left ventricular remodeling (LVR) and left ventricular hypertrophy (LVH) are particularly important, as both are closely associated with subsequent heart failure and poor prognosis [3, 4, 5]. Therefore, early identification of patients at high risk for developing LVR and LVH is of substantial clinical importance.

With the increasing use of advanced cardiac imaging techniques, myocardial strain analysis has become an important tool for the quantitative assessment of myocardial function [6, 7]. Among the available strain parameters, left ventricular global longitudinal strain (GLS), measured by two‐dimensional speckle‐tracking echocardiography (2D‐STE), is commonly used in clinical practice owing to its good feasibility and sensitivity [8, 9]. Available evidence indicates that GLS can detect subtle myocardial dysfunction even when conventional measures, such as left ventricular size or ejection fraction, remain preserved [10, 11]. Given that impaired myocardial deformation may occur before overt structural changes become apparent, GLS may have value in identifying patients at increased risk of subsequent LVR and LVH after AMI.

However, whether GLS measured early after PCI is associated with the subsequent development of LVR and LVH in patients with AMI remains unclear. Therefore, the present study aimed to investigate the association between early GLS measured after PCI and the subsequent risk of LVR and LVH, in order to provide evidence for early risk stratification in this population.

## 2 Methods

### 2.1 Study Design and Population

We prospectively enrolled patients with AMI who underwent emergency PCI within 24 h of symptom onset at the Jinshan Branch of Shanghai Sixth People's Hospital between March 2023 and October 2024. The protocol complied with the Declaration of Helsinki and was approved by the hospital's Medical Research Ethics Committee (jszxyy202209), which also waived the requirement for informed consent.

The key exclusion criteria were as follows: (1) patients aged under 18 years; (2) patients who did not complete speckle‐tracking echocardiography within 7 days after PCI; (3) patients who could not obtain satisfactory images by speckle‐tracking echocardiography; (4) patients who did not complete follow‐up; and (5) patients with severe arrhythmias, shock, a history of coronary artery bypass grafting, severe uncontrolled hypertension, congenital heart disease, severe infection, malignant tumors, dilated cardiomyopathy, cardiac hypertrophy (including hypertrophic cardiomyopathy and left ventricular hypertrophy), or myocarditis were excluded. Supporting Information S1: Figure S1 presents the study flow, including patient screening, exclusions, and the final analytic cohort.

### 2.2 Speckle‐Tracking Echocardiographic Image Acquisition and GLS Measurement

Echocardiography for GLS assessment was performed during the index hospitalization and before discharge, at a median of 4 days (IQR, 2–6) after PCI. Examinations were performed after clinical stabilization, although a fixed standardized post‐PCI time point was not prespecified. During the examination, patients were instructed to breathe calmly and lie in the left lateral decubitus position, with simultaneous electrocardiographic monitoring. Echocardiographic images were acquired by experienced sonographers using a Philips EPIQ 7 C cardiovascular ultrasound system (Philips Medical Systems, Best, the Netherlands). Standard grayscale images were obtained from the left ventricular apical two‐chamber, three‐chamber, and four‐chamber views during end‐expiratory breath‐hold over three consecutive cardiac cycles, with a frame rate of 50–80 frames/s. Offline strain analysis was performed using the TQMD program in QLAB software, version 10.8 (Philips Medical Systems, Best, The Netherlands). The software automatically tracked the endocardial and epicardial borders of the left ventricular myocardium throughout the cardiac cycle, and manual fine‐tuning was performed when necessary to ensure tracking accuracy. Peak systolic global longitudinal strain (GLS) of the entire left ventricle was then automatically calculated by the software, and the GLS value used in this study was the average of three consecutive cardiac cycles. In addition, to assess intra‐observer reproducibility, 20 patients were randomly selected, and GLS was measured twice by the same observer. Reproducibility was evaluated using the intraclass correlation coefficient (ICC), and the detailed results are shown in Supporting Information S1: Table S1.

### 2.3 Endpoint Definition

All patients were followed up by telephone, outpatient, or hospitalization after discharge. The primary outcome events were LVR and LVH. LVR was considered present when left ventricular end‐diastolic volume (LVEDV) increased by at least 20% on follow‐up echocardiography performed 6–12 months after the index examination [11]. LVH was defined as left ventricular mass index (LVMI) ≥ 115 g/m^2^ (males) or ≥ 95 g/m^2^ (females) [12]. Body surface area (BSA) (m^2^) = 0.0061 × height (cm) + 0.0128 × weight (kg) − 0.1529, LVM (g) = 1.04 × [(left ventricular end‐diastolic diameter + left ventricular end‐diastolic posterior wall thickness + interventricular septum thickness)^3^ − (left ventricular end‐diastolic diameter)^3^] × 0.8 + 0.6, LVMI (g/m^2^) = LVM (g)/BSA (m^2^) [13]. LVMI was assessed at baseline and again at follow‐up. Because patients with LVH at baseline were excluded, LVH identified during follow‐up was considered newly detected LVH.

### 2.4 Statistical Analyses

Continuous variables were summarized as mean ± standard deviation (SD) or median (interquartile range [IQR]) according to data distribution. The *t* test and Wilcoxon test were used for two‐group comparisons of normally and non‐normally distributed continuous variables, respectively, whereas one‐way ANOVA or the Kruskal–Wallis test was used for comparisons among three groups. Categorical variables are expressed as percentages and were compared with the *χ* ^2^ test or Fisher's exact test. Logistic regression analysis was used to explore the association of GLS with LVR and LVH. GLS was entered into the regression models as the original signed percentage rather than as an absolute value; therefore, a higher GLS value indicated a less negative strain value and more impaired left ventricular systolic function. The models were adjusted for age, diabetes, stroke, neutrophil, high‐sensitivity cardiac troponin T (hs‐TnT), symptom onset‐to‐balloon time (SBT), door‐to‐balloon time (D2B), N‐terminal pro‐brain natriuretic peptide (NT‐proBNP), left ventricular ejection fraction (LVEF), lymphocyte, uric acid, and stent diameter. These variables were selected based on their clinical relevance and potential associations with LVR and LVH. Restricted cubic spline (RCS) plots were drawn to calculate the dose‐response relationships of GLS with LVR and LVH. Receiver operating characteristic (ROC) analysis was used to assess how well GLS discriminated LVR and LVH. Subgroup analyses were further performed after stratification by age, sex, BMI, diabetes, and hypertension to examine the robustness of the associations between GLS and study outcomes. No formal a priori sample size or power calculation was performed, as this was an exploratory single‐center prospective observational study. All consecutive eligible patients during the study period were included. Given the limited number of outcome events, the study results should be interpreted cautiously.

Data analysis was performed using MedCalc version 20.0 and STATA version 15.0. A two‐sided significance level of *p* < 0.05 was adopted for all analyses.

## 3 Results

### 3.1 Basic Characteristics of Participants

A total of 131 patients with AMI were included in the final analysis. Their mean age was 67.86 ± 12.31 years, and 115 (87.79%) were male while 16 (12.21%) were female. Participants were categorized into tertiles according to GLS, and baseline characteristics were compared across the three groups. The results demonstrated that the percentage of smokers, history of previous stent implantation, percentage of coronary bifurcation lesions, time to D2B, percentage of LVR, and percentage of LVH were increased in participants in T3, representing more impaired LV systolic function compared to participants in T1 and T2 (all *p* < 0.05); and that stent diameter and LVEF were decreased (all *p* < 0.05). Additional details are presented in Table 1.

**Table 1:** Baseline characteristics of the study population according to GLS tertiles.

Variable	Total	T1	T2	T3	*p*
	(*n* = 131)	(*n* = 44)	(*n* = 43)	(*n* = 44)	
Age (year)	67.86 ± 12.31	65.36 ± 12.48	69.23 ± 12.19	69.02 ± 12.14	0.26
Gender (*n*, %)					0.58
Male	115 (87.79)	40 (90.91)	36 (83.72)	39 (88.64)	
Female	16 (12.21)	4 (9.09)	7 (16.28)	5 (11.36)	
BMI (kg/m^2^)	25.87 ± 3.45	25.91 ± 2.41	25.44 ± 4.25	26.27 ± 3.51	0.54
SBP (mmHg)	128.95 ± 17.92	126.11 ± 15.37	130.30 ± 20.58	130.45 ± 17.58	0.44
DBP (mmHg)	81.86 ± 12.28	80.36 ± 10.74	81.81 ± 14.13	83.41 ± 11.86	0.51
Heart rate (beats/min)	79.87 ± 12.11	79.45 ± 12.20	80.44 ± 11.08	79.72 ± 13.18	0.93
Current smoker (*n*, %)					0.03
No	65 (49.62)	15 (34.09)	27 (62.79)	23 (52.27)	
Yes	66 (50.38)	29 (65.91)	16 (37.21)	21 (47.73)	
Current drinker (*n*, %)					0.59
No	91 (69.47)	28 (63.64)	31 (72.09)	32 (72.73)	
Yes	40 (30.53)	16 (36.36)	12 (27.91)	12 (27.27)	
Hypertension (*n*, %)					0.49
No	73 (55.73)	27 (61.36)	21 (48.84)	25 (56.82)	
Yes	58 (44.27)	17 (38.64)	22 (51.16)	19 (43.18)	
Diabetes (*n*, %)					0.06
No	93 (70.99)	37 (84.09)	28 (65.12)	28 (63.64)	
Yes	38 (29.01)	7 (15.91)	15 (34.88)	16 (36.36)	
Stroke (*n*, %)					0.06
No	94 (71.76)	33 (75.00)	35 (81.40)	26 (59.09)	
Yes	37 (28.24)	11 (25.00)	8 (18.60)	18 (40.91)	
History of stent implantation (*n*, %)					0.03
No	123 (93.89)	38 (86.36)	41 (95.35)	44 (100.00)	
Yes	8 (6.11)	6 (13.64)	2 (4.65)	—	
White blood cell (×10^9^/L)	10.48 ± 2.65	11.05 ± 2.87	10.65 ± 2.62	9.74 ± 2.30	0.06
Neutrophil (×10^9^/L)	7.94 ± 2.52	8.21 ± 2.55	8.37 ± 2.44	7.25 ± 2.48	0.08
Lymphocyte (×10^9^/L)	1.59 ± 0.79	1.61 ± 0.73	1.42 ± 0.87	1.73 ± 0.76	0.19
Hemoglobin (g/L)	147.69 ± 16.86	151.94 ± 15.65	147.21 ± 16.87	143.91 ± 17.42	0.08
Platelet (×10^9^/L)	211.14 ± 51.08	216.36 ± 46.02	210.32 ± 55.73	206.72 ± 51.87	0.67
Hs‐CRP (mg/L)	9.04 ± 9.91	9.58 ± 9.36	9.02 ± 10.66	8.51 ± 9.90	0.88
eGFR (ml/min/1.73m^2^)	109.95 ± 12.88	110.35 ± 13.29	111.85 ± 11.72	107.69 ± 13.49	0.31
Total cholesterol (mmol/L)	4.58 ± 1.02	4.46 ± 0.93	4.55 ± 1.07	4.74 ± 1.07	0.43
Triglyceride (mmol/L)	1.56 ± 0.77	1.63 ± 0.77	1.43 ± 0.80	1.63 ± 0.74	0.39
HDL‐C (mmol/L)	1.00 ± 0.24	0.97 ± 0.25	1.01 ± 0.23	1.01 ± 0.25	0.65
LDL‐C (mmol/L)	2.90 ± 0.91	2.75 ± 0.83	2.89 ± 0.93	3.05 ± 0.96	0.30
Lipoprotein (a) (mg/dL)	235.81 ± 147.16	212.33 ± 109.89	216.92 ± 153.41	277.75 ± 166.40	0.07
Uric acid (umol/L)	302.12 ± 82.26	313.26 ± 85.29	291.84 ± 76.01	301.02 ± 85.46	0.48
glycohemoglobin (%)	6.73 ± 1.43	6.55 ± 1.37	6.82 ± 1.50	6.81 ± 1.45	0.62
CKMB (ng/mL)	103.97 ± 75.06	117.58 ± 82.54	85.73 ± 56.04	108.19 ± 81.31	0.13
hs‐cTnT (ng/mL)	3.61 ± 3.24	3.51 ± 3.35	3.74 ± 3.39	3.59 ± 3.05	0.95
NT‐proBNP (pg/mL)	1460.44 ± 1311.49	1255.27 ± 1165.79	1457.16 ± 1430.98	1668.82 ± 1324.12	0.34
D‐dimer (mg/L)	0.30 ± 0.18	0.28 ± 0.18	0.32 ± 0.21	0.29 ± 0.17	0.60
Pre‐AP (*n*, %)					0.08
No	96 (73.28)	34 (77.27)	35 (81.40)	27 (61.36)	
Yes	35 (26.72)	10 (22.73)	8 (18.60)	17 (38.64)	
Killip class (*n*, %)					0.09
I	1 (0.76)	1 (2.27)	—	—	
II	121 (92.37)	38 (86.36)	40 (93.02)	43 (97.73)	
III	7 (5.34)	5 (11.36)	1 (2.33)	1 (2.27)	
IV	2 (1.53)	—	2 (4.65)	—	
SBT (min)	255.37 ± 144.20	212.03 ± 109.89	252.94 ± 142.73	301.08 ± 163.77	0.01
D2B (min)	66.00 ± 25.38	58.79 ± 23.91	61.62 ± 19.81	77.51 ± 28.01	< 0.001
Diameter of stent (mm)	3.06 ± 0.42	3.25 ± 0.43	3.00 ± 0.39	2.93 ± 0.39	< 0.001
Length of stent (mm)	27.82 ± 10.01	28.07 ± 10.12	27.49 ± 10.08	27.91 ± 10.06	0.96
Multivascular disease (*n*, %)					0.37
No	43 (32.82)	17 (38.64)	15 (34.88)	11 (25.00)	
Yes	88 (67.18)	27 (61.36)	28 (65.12)	33 (75.00)	
LAD (*n*, %)					0.38
No	65 (49.62)	25 (56.82)	18 (41.86)	22 (50.00)	
Yes	66 (50.38)	19 (43.18)	25 (58.14)	22 (50.00)	
LCX (*n*, %)					0.29
No	114 (87.02)	36 (81.82)	40 (93.02)	38 (86.36)	
Yes	17 (12.98)	8 (18.18)	3 (6.98)	6 (13.64)	
RCA (*n*, %)					0.84
No	81 (61.83)	26 (59.09)	28 (65.12)	27 (61.36)	
Yes	50 (38.17)	18 (40.91)	15 (34.88)	17 (38.64)	
Calcified lesions (*n*, %)					0.12
No	125 (95.42)	44 (100.00)	39 (90.70)	42 (95.45)	
Yes	6 (4.58)	—	4 (9.30)	2 (4.55)	
Proximal lesions (*n*, %)					0.72
No	55 (41.98)	20 (45.45)	16 (37.21)	19 (43.18)	
Yes	76 (58.02)	24 (54.55)	27 (62.79)	25 (56.82)	
Bifurcation lesion (*n*, %)					0.02
No	112 (85.50)	43 (97.73)	34 (79.07)	35 (79.55)	
Yes	19 (14.50)	1 (2.27)	9 (20.93)	9 (20.45)	
No reflow (*n*, %)					0.95
No	96 (73.28)	32(72.73)	31 (72.09)	33 (75.00)	
Yes	35 (26.72)	12(27.27)	12 (27.91)	11 (25.00)	
Nitroprusside (*n*, %)					0.35
No	77 (58.78)	28 (63.64)	27 (62.79)	22 (50.00)	
Yes	54 (41.22)	16 (36.36)	16 (37.21)	22 (50.00)	
Tirofiban (*n*, %)					0.45
No	103 (78.63)	35 (79.55)	36 (83.72)	32 (72.73)	
Yes	28 (21.37)	9 (20.45)	7 (16.28)	12 (27.27)	
Atropine (*n*, %)					0.29
No	120 (91.60)	38 (86.36)	41 (95.35)	41 (93.18)	
Yes	11 (8.40)	6 (13.64)	2 (4.65)	3 (6.82)	
Dopamine (*n*, %)					0.60
No	123 (93.89)	40 (90.91)	41 (95.35)	42 (95.45)	
Yes	8 (6.11)	4 (9.09)	2 (4.65)	2 (4.55)	
Stent thrombosis (*n*, %)					0.18
No	121 (92.37)	38 (86.36)	41 (95.35)	42 (95.45)	
Yes	10 (7.63)	6 (13.64)	2 (4.65)	2 (4.55)	
Temporary cardiac pacing (*n*, %)					0.36
No	130 (99.24)	44 (100.00)	42 (97.67)	44 (100.00)	
Yes	1 (0.76)	—	1 (2.33)	—	
LVEF (%)	43.21 ± 9.97	46.07 ± 10.36	43.28 ± 10.16	40.30 ± 8.68	0.02
LVR (*n*, %)					0.01
No	102 (77.86)	38 (86.36)	37 (86.05)	27 (61.36)	
Yes	29 (22.14)	6 (13.64)	6 (13.95)	17 (38.64)	
LVH (*n*, %)					< 0.001
No	94 (71.76)	40 (90.91)	36 (83.72)	18 (40.91)	
Yes	37 (28.24)	4 (9.09)	7 (16.28)	26 (59.09)	

Abbreviations: T1: < −12.973%; T2: −12.973% to −11.207%; T3: > −11.207%. BMI, body mass index; CKMB, creatine kinase MB isoenzyme; D2B, door‐to‐balloon time; DBP, diastolic blood pressure; eGFR, estimated glomerular filtration rate; GLS, global longitudinal strain; hs‐cTnT, high‐sensitivity cardiac troponin T; hs‐CRP, high‐sensitivity C‐reactive protein; HDL‐C, high density lipoprotein‐cholesterol; LDL‐C, low density lipoprotein‐cholesterol; LAD, left anterior descending; LCX, left circumflex branch; LVEF, left ventricular ejection fraction; LVR, left ventricular remodeling; LVH, left ventricular hypertrophy; NT‐proBNP, N‐terminal pro‐brain natriuretic peptide; pre‐AP, pre‐infarction angina; RCA, right coronary artery; SBT, symptom onset‐to‐balloon time; SBP, systolic blood pressure.

According to the occurrence of LVH during follow‐up, participants were classified into the LVH group (*n* = 37, 28.24%) and the non‐LVH group (*n* = 94, 71.76%). Compared with the non‐LVH group, patients in the LVH group had a higher percentage of history of diabetes, longer D2B time and SBT (all *p* < 0.05), and lower stent diameter, LVEF and GLS (all *p* < 0.05). Similarly, participants were classified into the LVR group (*n* = 29, 22.14%) and the non‐LVR group (*n* = 102, 77.86%) based on whether LVR developed during follow‐up. Higher lymphocyte counts, hs‐TnT, and NT‐proBNP levels and lower stent diameter and GLS were observed in patients with LVR than in those without LVR (all *p* < 0.05). Table 2 presents the detailed results.

**Table 2:** Baseline characteristics of the study population grouped by LVH and LVR status.

Variable	Non‐LVH	LVH	*p*	Non‐LVR	LVR	*p*
	(*n* = 94)	(*n* = 37)		(*n* = 102)	(*n* = 29)	
Age (year)	67.29 ± 11.78	69.32 ± 13.61	0.43	66.63 ± 11.71	72.21 ± 13.54	0.05
Gender (*n*, %)			0.55			0.50
Male	81 (86.17)	34 (91.89)		88 (86.27)	27 (93.10)	
Female	13 (13.83)	3 (8.11)		14 (13.73)	2 (6.90)	
BMI (kg/m^2^)	25.93 ± 3.38	25.73 ± 3.68	0.78	25.68 ± 3.37	26.54 ± 3.72	0.27
SBP (mmHg)	129.04 ± 18.62	128.70 ± 16.26	0.92	128.80 ± 18.40	129.45 ± 16.44	0.86
DBP (mmHg)	82.27 ± 12.81	80.84 ± 10.90	0.52	82.07 ± 12.69	81.14 ± 10.86	0.70
Heart rate (beats/min)	79.50 ± 11.36	80.80 ± 13.97	0.61	80.00 ± 11.68	79.41 ± 13.72	0.84
Current smoker (*n*, %)			0.22			0.43
No	43 (45.74)	22 (59.46)		53 (51.96)	12 (41.38)	
Yes	51 (54.26)	15 (40.54)		49 (48.04)	17 (58.62)	
Current drinker (*n*, %)			0.61			0.87
No	67 (71.28)	24 (64.86)		70 (68.63)	21 (72.41)	
Yes	27 (28.72)	13 (35.14)		32 (31.37)	8 (27.59)	
Hypertension (*n*, %)			0.26			0.78
No	49 (52.13)	24 (64.86)		58 (56.86)	15 (51.72)	
Yes	45 (47.87)	13 (35.14)		44 (43.14)	14 (48.28)	
Diabetes (*n*, %)			0.04			0.97
No	72 (76.60)	21 (56.76)		73 (71.57)	20 (68.97)	
Yes	22 (23.40)	16 (43.24)		29 (28.43)	9 (31.03)	
Stroke (n, %)			0.08			0.89
No	72 (76.60)	22 (59.46)		74 (72.55)	20 (68.97)	
Yes	22 (23.40)	15 (40.54)		28 (27.45)	9 (31.03)	
History of stent implantation (*n*, %)			0.54			0.81
No	87 (92.55)	36 (97.30)		95 (93.14)	28 (96.55)	
Yes	7 (7.45)	1 (2.70)		7 (6.86)	1 (3.45)	
White blood cell (×10^9^/L)	10.69 ± 2.61	9.92 ± 2.70	0.14	10.58 ± 2.56	10.13 ± 2.97	0.47
Neutrophil (×10^9^/L)	8.20 ± 2.56	7.29 ± 2.33	0.05	8.10 ± 2.40	7.40 ± 2.89	0.24
Lymphocyte (×10^9^/L)	1.57 ± 0.80	1.63 ± 0.78	0.68	1.51 ± 0.77	1.88 ± 0.81	0.03
Hemoglobin (g/L)	148.75 ± 16.96	144.99 ± 16.53	0.25	147.69 ± 16.90	147.70 ± 17.03	> 0.99
Platelet (×109/L)	215.22 ± 48.68	200.79 ± 56.10	0.17	214.07 ± 51.43	200.84 ± 49.33	0.21
Hs‐CRP (mg/L)	8.11 ± 9.23	11.40 ± 11.26	0.12	9.23 ± 10.01	8.35 ± 9.72	0.67
eGFR (ml/min/1.73 m^2^)	110.92 ± 12.53	107.48 ± 13.60	0.19	110.66 ± 12.98	107.44 ± 12.43	0.23
Total cholesterol (mmol/L)	4.55 ± 1.07	4.67 ± 0.91	0.51	4.57 ± 1.07	4.61 ± 0.87	0.86
Triglyceride (mmol/L)	1.51 ± 0.75	1.70 ± 0.81	0.22	1.53 ± 0.75	1.67 ± 0.85	0.45
HDL‐C (mmol/L)	1.02 ± 0.24	0.95 ± 0.24	0.14	1.00 ± 0.24	1.00 ± 0.24	0.92
LDL‐C (mmol/L)	2.85 ± 0.94	3.03 ± 0.83	0.28	2.89 ± 0.94	2.93 ± 0.79	0.84
Lipoprotein (a) (mg/dL)	232.87 ± 143.06	243.29 ± 158.91	0.73	234.88 ± 142.65	239.07 ± 164.71	0.9
Uric acid (umol/L)	297.13 ± 81.40	314.80 ± 84.19	0.28	295.06 ± 83.56	326.97 ± 73.55	0.05
glycohemoglobin (%)	6.61 ± 1.32	7.02 ± 1.67	0.19	6.75 ± 1.47	6.64 ± 1.34	0.70
CKMB (ng/mL)	97.58 ± 70.67	120.21 ± 84.03	0.15	100.17 ± 69.89	117.35 ± 91.09	0.35
hs‐cTnT (ng/mL)	3.30 ± 3.33	4.39 ± 2.90	0.07	3.22 ± 3.23	5.00 ± 2.91	0.01
NT‐proBNP (pg/mL)	1324.78 ± 1324.41	1805.10 ± 1228.68	0.05	1243.94 ± 1246.43	2221.92 ± 1269.01	< 0.001
D‐dimer (mg/L)	0.30 ± 0.18	0.30 ± 0.19	0.88	0.31 ± 0.19	0.25 ± 0.15	0.10
Pre‐AP (*n*, %)			0.11			> 0.99
No	73 (77.66)	23 (62.16)		75 (73.53)	21 (72.41)	
Yes	21 (22.34)	14 (37.84)		27 (26.47)	8 (27.59)	
Killip class (*n*, %)			0.67			0.20
I	1 (1.06)	—		—	1 (3.45)	
II	86 (91.49)	35 (94.59)		95 (93.14)	26 (89.66)	
III	6 (6.38)	1 (2.70)		6 (5.88)	1 (3.45)	
IV	1 (1.06)	1 (2.70)		1 (0.98)	1 (3.45)	
SBT (min)	237.57 ± 134.76	300.57 ± 158.88	0.04	256.87 ± 144.89	250.10 ± 144.13	0.82
D2B (min)	61.60 ± 23.10	77.20 ± 27.73	0.01	64.58 ± 24.80	71.01 ± 27.18	0.26
Diameter of stent (mm)	3.11 ± 0.42	2.93 ± 0.42	0.03	3.10 ± 0.43	2.91 ± 0.37	0.03
Length of stent (mm)	27.29 ± 10.11	29.19 ± 9.74	0.32	27.91 ± 9.94	27.52 ± 10.43	0.86
Multivascular disease (*n*, %)			0.79			0.65
No	32 (34.04)	11 (29.73)		35 (34.31)	8 (27.59)	
Yes	62 (65.96)	26 (70.27)		67 (65.69)	21 (72.41)	
LAD (*n*, %)			0.66			0.64
No	45 (47.87)	20 (54.05)		49 (48.04)	16 (55.17)	
Yes	49 (52.13)	17 (45.95)		53 (51.96)	13 (44.83)	
LCX (*n*, %)			0.69			> 0.99
No	83 (88.30)	31 (83.78)		89 (87.25)	25 (86.21)	
Yes	11 (11.70)	6 (16.22)		13 (12.75)	4 (13.79)	
RCA (*n*, %)			> 0.99			0.54
No	58 (61.70)	23 (62.16)		65 (63.73)	16 (55.17)	
Yes	36 (38.30)	14 (37.84)		37 (36.27)	13 (44.83)	
Calcified lesions (*n*, %)			> 0.99			> 0.99
No	90 (95.74)	35 (94.59)		97 (95.10)	28 (96.55)	
Yes	4 (4.26)	2 (5.41)		5 (4.90)	1 (3.45)	
Proximal lesions (*n*, %)			0.24			0.32
No	36 (38.30)	19 (51.35)		40 (39.22)	15 (51.72)	
Yes	58 (61.70)	18 (48.65)		62 (60.78)	14 (48.28)	
Bifurcation lesion (*n*, %)			0.24			0.31
No	83 (88.30)	29 (78.38)		85 (83.33)	27 (93.10)	
Yes	11 (11.70)	8 (21.62)		17 (16.67)	2 (6.90)	
No reflow (*n*, %)			> 0.99			0.72
No	69 (73.40)	27 (72.97)		76 (74.51)	20 (68.97)	
Yes	25 (26.60)	10 (27.03)		26 (25.49)	9 (31.03)	
Nitroprusside (*n*, %)			0.09			0.82
No	60 (63.83)	17 (45.95)		61 (59.80)	16 (55.17)	
Yes	34 (36.17)	20 (54.05)		41 (40.20)	13 (44.83)	
Tirofiban (*n*, %)			0.78			> 0.99
No	75 (79.79)	28 (75.68)		80 (78.43)	23 (79.31)	
Yes	19 (20.21)	9 (24.32)		22 (21.57)	6 (20.69)	
Atropine (*n*, %)			0.26			0.48
No	84 (89.36)	36 (97.30)		92 (90.20)	28 (96.55)	
Yes	10 (10.64)	1 (2.70)		10 (9.80)	1 (3.45)	
Dopamine (*n*, %)			> 0.99			0.81
No	88 (93.62)	35 (94.59)		95 (93.14)	28 (96.55)	
Yes	6 (6.38)	2 (5.41)		7 (6.86)	1 (3.45)	
Stent thrombosis (*n*, %)			> 0.99			0.17
No	87 (92.55)	34 (91.89)		92 (90.20)	29 (100.00)	
Yes	7 (7.45)	3 (8.11)		10 (9.80)	—	
Temporary cardiac pacing (*n*, %)			> 0.99			> 0.99
No	93 (98.94)	37 (100.00)		101 (99.02)	29 (100.00)	
Yes	1 (1.06)	—		1 (0.98)	—	
LVEF (%)	44.95 ± 10.39	38.81 ± 7.21	< 0.001	44.13 ± 9.66	40.00 ± 10.54	0.07
GLS (%)	−12.74 ± 2.23	−10.75 ± 1.75	< 0.001	−12.56 ± 2.18	−10.86 ± 2.16	< 0.001

Abbreviations: BMI, body mass index; CKMB, creatine kinase MB isoenzyme; DBP, diastolic blood pressure; D2B, door‐to‐balloon time; eGFR, estimated glomerular filtration rate; hs‐cTnT, high‐sensitivity cardiac troponin T; hs‐CRP, high‐sensitivity C‐reactive protein; HDL‐C, high density lipoprotein‐cholesterol; GLS, global longitudinal strain; LAD, left anterior descending; LCX, left circumflex branch; LDL‐C, low density lipoprotein‐cholesterol; LVH, left ventricular hypertrophy; LVR, left ventricular remodeling; NT‐proBNP, N‐terminal pro‐brain natriuretic peptide; SBP, systolic blood pressure; pre‐AP, pre‐infarction angina; RCA, right coronary artery; LVEF, left ventricular ejection fraction; SBT, symptom onset‐to‐balloon time.

### 3.2 Association of GLS With LVR and LVH in Patients With AMI

**Figure 1:**
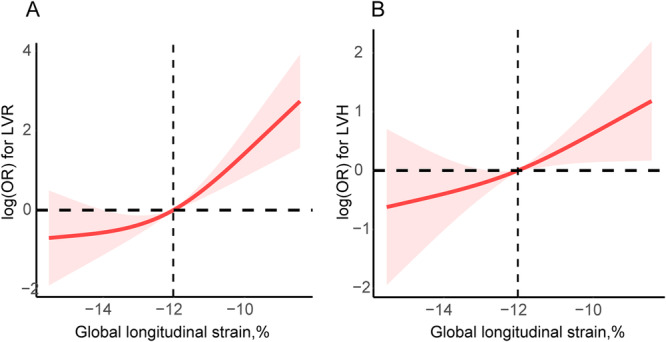
Restricted cubic spline curves of the relationships between GLS levels and LVR (A) and LVH (B). GLS, global longitudinal strain; LVR, left ventricular remodeling; LVH, left ventricular hypertrophy.

**Figure 2:**
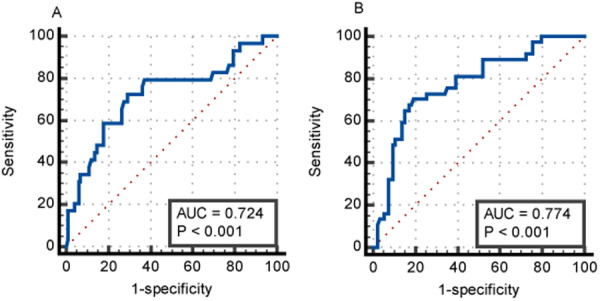
ROC curves of GLS for discriminating LVR (A) and LVH (B). GLS, global longitudinal strain; LVR, left ventricular remodeling; LVH, left ventricular hypertrophy; ROC, receiver operating characteristic.

As shown in Table 3, in the unadjusted model, impaired GLS was significantly associated with higher risks of both LVR (OR = 1.49, 95% CI: 1.19–1.88, *p* < 0.01) and LVH (OR = 1.65, 95% CI: 1.31–2.09, *p* < 0.01). After further adjustment in Model 2, GLS remained significantly associated with LVR, whereas the association between GLS and LVH was attenuated. Further analyses based on GLS tertiles showed that, compared with patients in T1, representing better preserved LV systolic function, those in T3, representing more impaired LV systolic function, had significantly higher risks of LVR (OR = 7.51, 95% CI: 1.41–39.96, *p* = 0.02) and LVH (OR = 6.15, 95% CI: 1.40–27.04, *p* = 0.02). RCS analysis revealed a linear relationship between GLS and both LVR (*P* for nonlinearity = 0.152) (Figure 1A) and LVH (*P* for nonlinearity = 0.714) (Figure 1B). As shown in Figure 2, the AUC of GLS for discriminating LVR was 0.724 (95% CI: 0.640–0.799), with an optimal cut‐off point of −11.54%, a specificity of 70.59%, and a sensitivity of 72.41%. The AUC of GLS for discriminating LVH was 0.774 (95% CI: 0.693–0.843), with an optimal cut‐off point of −11.22%, a specificity of 80.85%, and a sensitivity of 70.27%.

**Table 3:** Logistic regression analysis of the associations of GLS with LVR and LVH in patients with AMI.

Outcome	Model 1		Model 2	
	OR (95% CI)	*p*	OR (95% CI)	*p*
LVR ~ GLS				
GLS	1.49 (1.19,1.88)	< 0.001	1.69 (1.19, 2.40)	0.003
Tertiles of GLS				
T1	Ref.		Ref.	
T2	1.03 (0.30, 3.47)	0.97	0.96 (0.19, 4.86)	0.96
T3	3.99 (1.39, 11.43)	0.01	7.51 (1.41, 39.96)	0.02
*P* for trend		0.01		0.01
LVH ~ GLS				
GLS	1.65 (1.31, 2.09)	< 0.001	1.31 (0.98, 1.77)	0.07
Tertiles of GLS				
T1	Ref.		Ref.	
T2	1.94 (0.53, 7.19)	0.32	1.1 (0.24, 5.06)	0.91
T3	14.44 (4.39, 47.51)	< 0.001	6.15 (1.40, 27.04)	0.02
*P* for trend		< 0.001		0.01

*Note:* For continuous analysis, ORs were calculated per 1% increase in GLS. T1 represents better preserved LV systolic function, whereas T3 represents more impaired LV systolic function. No covariates were adjusted. Model 2 adjusted for age, diabetes, stroke, neutrophil, high‐sensitivity cardiac troponin T, symptom onset‐to‐balloon time, door‐to‐balloon time, N‐terminal pro‐brain natriuretic peptide, left ventricular ejection fraction, lymphocyte, uric acid, and stent diameter.

Abbreviations: AMI, acute myocardial infarction; CI: confidence interval; GLS, global longitudinal strain; LVR, left ventricular remodeling; LVH, left ventricular hypertrophy; OR: odds ratio.

### 3.3 Subgroup Analysis and Interaction

**Figure 3:**
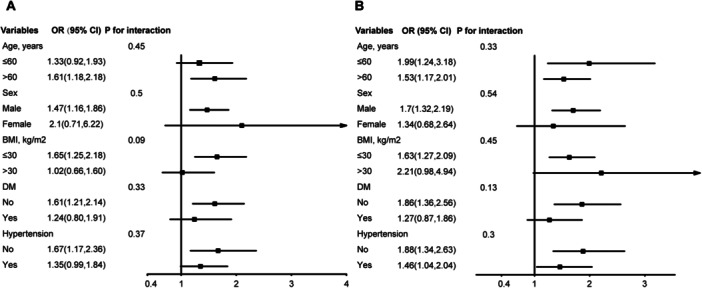
Forest plots showing ORs and 95% CIs for the associations of LVR (A) and LVH (B) across subgroups. CIs, confidence intervals; LVR, left ventricular remodeling; LVH, left ventricular hypertrophy; ORs, odds ratios.

Subgroup analyses stratified by age, sex, BMI, diabetes status, and hypertension consistently showed that impaired GLS was associated with an increased risk of both LVR (Figure 3A) and LVH (Figure 3B) across the different subgroups. No significant interactions were observed between GLS and any stratification variable for either outcome (all *p* for interaction > 0.05), indicating that the associations of impaired GLS with LVR and LVH were generally stable across subgroups.

## 4 Discussion

LVR and LVH following AMI are important structural abnormalities that are closely associated with adverse prognosis and the subsequent development of heart failure [12, 13, 14]. GLS has increasingly been used to characterize subtle myocardial dysfunction in different clinical settings [15, 16]. Previous studies have shown that reduced longitudinal strain is associated with cardiovascular disease and poor prognosis [17, 18, 19, 20, 21, 22]. In this small single‐center prospective observational study, impaired GLS measured within 7 days after primary PCI was associated with subsequent LVR and LVH in patients with AMI. These findings suggest that early GLS assessment may help identify patients at increased risk of post‐infarction structural ventricular changes.

In the present study, more impaired GLS was independently associated with a higher likelihood of subsequent LVR, in line with the observations reported by Juan Lacalzada et al. [23]. In that study, LVR occurred in 38 of 97 patients with AMI during a mean follow‐up period of 22.8 ± 12.3 months, and GLS independently predicted this outcome [23]. Similar findings were also reported by Sung Mok Kim et al. in patients with ST‐segment elevation myocardial infarction [24]. In addition, our adjusted model suggested that GLS retained its predictive value for LVR even after accounting for conventional clinical factors, suggesting that GLS may provide additional clinically relevant information beyond these factors [25]. This finding is also supported by studies in other populations. Vos et al. reported that in a cohort of 162 patients with acute myocarditis followed for a mean of 5.5 years, impaired GLS independently predicted major adverse cardiovascular events [26]. Xie et al. also showed that lower GLS was an independent predictor of MACE in patients with end‐stage renal disease within 12 months [27].

Several mechanisms may explain the association between impaired GLS and LVR after AMI. When AMI occurs, myocardial blood flow is abruptly reduced, leading to cardiomyocyte injury and damage to myocardial contractile proteins. The subendocardial layer is usually affected first, followed by the mid‐myocardial and even epicardial layers [28, 29]. Because longitudinal myocardial fibers are predominantly distributed in the subendocardium, impairment of these fibers results in reduced longitudinal deformation and regional mechanical asynchrony, making GLS more sensitive than circumferential or radial strain in detecting early myocardial dysfunction [30]. In addition, post‐infarction inflammatory and fibrotic responses may contribute to progressive ventricular remodeling [31].

In addition to LVR, our results also suggested that impaired GLS was associated with an increased risk of LVH. Although the fully adjusted association was attenuated, tertile and dose‐response analyses still supported a relationship between GLS and LVH. The underlying mechanism is not fully understood, but may involve abnormalities in myocardial wall stress, neuroendocrine activation, and metabolic remodeling. Reduced GLS may reflect injury to subendocardial longitudinal fibers, which could contribute to abnormal ventricular wall stress distribution and subsequent compensatory fibrosis and hypertrophy [32]. In addition, decreased GLS may be accompanied by elevated left ventricular filling pressure, which may activate neurohormonal pathways such as the renin‐angiotensin‐aldosterone system and thereby contribute to cardiomyocyte hypertrophy and collagen deposition [33, 34]. Metabolic remodeling and impaired mitochondrial homeostasis after ischemic injury may also be involved in this process [35, 36]. However, given that the association between GLS and LVH was attenuated after multivariable adjustment, this finding should be interpreted with caution. The ROC‐derived GLS cut‐off values for predicting LVR and LVH in our study were −11.54% and −11.22%, respectively. These thresholds are slightly less negative than those reported in some previous studies of post‐infarction adverse remodeling, in which optimal GLS cut‐off values were generally around −12.46% to −12.8% [23, 37, 38]. Nevertheless, they remain within a broadly similar impaired range. Several factors may account for this difference, including the early timing of GLS assessment within 7 days after PCI, platform‐ and software‐related variability in GLS measurement, and the modest sample size of this single‐center study. In addition, because published data on GLS thresholds for predicting newly detected LVH after AMI are limited, the LVH cut‐off identified in our study should be regarded as a preliminary finding and not as an established clinical threshold.

The present study has several clinical implications. Because GLS can detect subtle myocardial dysfunction earlier than conventional echocardiographic indices, early GLS assessment after PCI may help identify patients at high risk of adverse ventricular structural changes. In addition, the linear dose‐response relationship observed in our analysis supports the potential clinical utility of GLS in risk stratification. Subgroup analyses further showed that the association between impaired GLS and the risks of LVR and LVH was generally stable across different patient subgroups, which further supports the robustness of our findings.

Several limitations should be acknowledged. First, the single‐center design and modest sample size may limit the generalizability of our findings. Second, only patients who met the predefined inclusion criteria and had complete echocardiographic and follow‐up data were included in the final analysis, and thus selection bias cannot be completely excluded. Third, as the duration of follow‐up ranged from 6 to 12 months, the current study was not able to evaluate longer‐term clinical outcomes. Fourth, although multiple potential confounders were adjusted for, some relevant factors, including infarct location, medication use after discharge, and more direct measures of infarct size, were not available or were not included in the final adjusted models; therefore, residual confounding remains possible. Fifth, because all GLS analyses were performed by a single observer, inter‐observer reproducibility could not be assessed in the present study. Finally, the modest sample size, limited number of outcome events, and number of covariates included in the adjusted models may have reduced statistical power and increased the risk of overfitting. Accordingly, the findings should be interpreted with caution and require validation in larger multicenter studies.

## 5 Conclusions

Early impairment of GLS in patients with AMI after PCI was associated with subsequent LVR and LVH. These findings suggest that GLS may be useful for early risk stratification after AMI, although validation in larger multicenter studies is needed.

## Author Contributions

**Bing Wang:** writing – review and editing, writing – original draft, methodology, visualization, conceptualization. **Hong Yao:** methodology and formal analysis. **Zhijian Zhu:** writing – original draft, data curation, software. **Zhigang Lu:** investigation, validation. **Yesheng Pan:** writing – review and editing, funding acquisition, project administration, resources, and supervision.

## Ethics Statement

This study was conducted in accordance with the Declaration of Helsinki and approved by the Medical Research Ethics Committee of the Jinshan Branch of Shanghai Sixth People's Hospital (approval number: jszxyy202209). The need for informed consent was waived by the Medical Research Ethics Committee of the Jinshan Branch of Shanghai Sixth People's Hospital.

## Consent

The authors have nothing to report.

## Conflicts of Interest

The authors declare no conflicts of interest.

## Transparency Statement

Yesheng Pan confirms that this manuscript is an honest, accurate, and transparent account of the study being reported; that no important aspects of the study have been omitted; and that any discrepancies from the study as planned have been explained.

## Supporting information

Supporting File

## Data Availability

The datasets used and/or analyzed during the current study are available from the corresponding author (felixpan7519@163. com) upon reasonable request.
