# Supplementary material for: Association of Global Longitudinal Strain With Left Ventricular Remodeling and Left Ventricular Hypertrophy in Patients With Acute Myocardial Infarction After PCI: A Small Single‐Center Prospective Observational Study
Source: Health Sci Rep. 2026 Jun 28;9(7):e72716. doi: 10.1002/hsr2.72716 (PMC13311305; doi:10.1002/hsr2.72716)
Supplement: Supplementary file 1 — Supporting File [file HSR2-9-e72716-s001.docx]

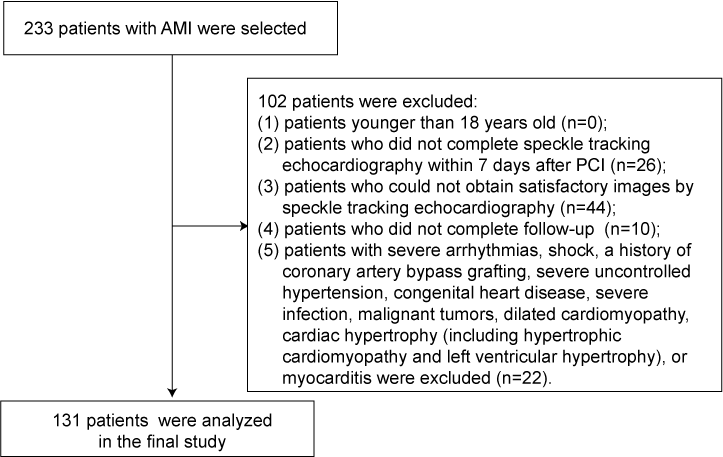


**Figure S1.** The flowchart of this study.

**Table S1.** Intra-observer reproducibility of GLS measurements in 20 randomly selected patients.

| Strain Parameters | ICC | 95% CI |
| --- | --- | --- |
| GLS | 0.857 | 0.676 - 0.941 |

ICC was calculated using a two-way mixed-effects model with absolute agreement for single measurements. GLS was measured twice by the same observer. The results indicate good intra-observer reproducibility. **Abbreviations:** GLS, global longitudinal strain; ICC, intraclass correlation coefficient; CI, confidence interval.
